# The relationship between major depression, attention-deficit hyperactivity disorder and coronary artery disease: A two-sample Mendelian randomization analysis

**DOI:** 10.1097/MD.0000000000043188

**Published:** 2025-10-17

**Authors:** Xiaochen Zhang, Lei Zhang, Suxia Huo, Yan Lu, Xiaokai Hua, Xiaofei Zhong

**Affiliations:** a Shandong University of Traditional Chinese Medicine, Jinan, P.R. China; b Qingdao Shibei District Shuangtao Hospital, Qingdao, P.R. China; c Second Affiliated Hospital of Shandong University of Traditional Chinese Medicine, Jinan, P.R. China.

**Keywords:** attention-deficit hyperactivity disorder, coronary artery disease, major depression, Mendelian randomization, risk factors

## Abstract

Coronary artery disease (CAD) constitutes a principal cause of global morbidity and mortality. Studies imply a connection between mental health disorders, especially major depression (MD) and attention-deficit hyperactivity disorder (ADHD), and the risk of CAD. To investigate the causal influence of genetic susceptibility to MD and attention-deficit/hyperactivity disorder (ADHD) on the risk of CAD, summary-level data from genome-wide association studies involving individuals of European descent were utilized. This analysis identified 11 single-nucleotide polymorphisms (SNPs) associated with MD, 60 SNPs linked to ADHD, and 10 SNPs related to CAD as instrumental variables. The inverse variance weighted method was employed for causal estimation, complemented by sensitivity analyses using MR-Egger regression and the weighted median estimator. A positive causal relationship was identified between MD, attention-deficit/hyperactivity disorder (ADHD), and the risk of CAD [MD: Odds Ratio (OR): 42.66, 95% Confidence Interval (CI): 7.55–241.2; ADHD: OR: 1.055, 95% CI: 1.006–1.106]. No significant causal association was observed between obesity and ADHD. In the multivariable Mendelian randomization (MVMR) analysis, the causal effect of ADHD on CAD was found to be diminished (OR: 1.005, 95% CI: 0.998–1.020), while the impact of body mass index on CAD remained stable (OR: 1.557, 95% CI: 1.459–1.661). This Mendelian randomization study reveals the lack of a consistent association among MD, ADHD, and CAD, suggesting a causal relationship and bidirectional effects between ADHD and obesity.

## 1. Introduction

Coronary artery disease (CAD) remains one of the leading causes of morbidity and mortality worldwide. Understanding the underlying risk factors and potential causal pathways is crucial for developing effective prevention and treatment strategies.^[1]^ Recently, there has been growing interest in the potential link between mental health disorders and cardiovascular diseases. Among these, major depression (MD) and attention-deficit hyperactivity disorder (ADHD) have been of particular concern due to their high prevalence and significant impact on quality of life.^[2,3]^

MD is a common mental health disorder characterized by persistent feelings of sadness, loss of interest, and a range of physical and emotional problems.^[4]^ It affects millions of people globally and has been associated with an elevated risk of developing CAD.^[5]^ The mechanisms that may underlie this association include behavioral factors such as physical inactivity and poor dietary habits, as well as biological pathways involving inflammation, autonomic dysfunction, and hypercoagulability.^[6–8]^ Haleh Ayatollahi conducted a study to identify risk factors associated with CAD utilizing a support vector machine model.^[9]^ The findings revealed that the incidence of CAD is positively correlated with both age and weight.^[10]^ Similarly, another investigation identified significant correlations between age, male gender, and smoking habits with the prevalence of CAD.^[11]^ A predictive system for heart attacks was proposed, which incorporated various variables including gender, age, type of chest pain, heart rate, cholesterol levels, smoking status, blood glucose levels, blood pressure readings, dietary habits, and alcohol consumption.^[12]^ Furthermore, results from a cohort study indicated that work-related stress elevates the risk of cardiovascular diseases by approximately 40%, while an increase in workload can double this risk.^[13]^

ADHD, a neurodevelopmental condition marked by persistent patterns of inattention, hyperactivity, and impulsivity, is traditionally considered a childhood disorder but often persists into adulthood^[14]^ ADHD is associated with various comorbidities, including cardiovascular diseases. While the mechanisms linking ADHD to CAD are less well understood, they may involve shared genetic factors, lifestyle behaviors, and stress-related physiological responses.^[10,11]^ Growing evidence from case-control and cohort studies suggests that ADHD is linked to a range of physical health conditions, including obesity, type 2 diabetes mellitus, and hypertension – all known risk factors for cardiovascular disease.^[15]^ Furthermore, ADHD has been associated with asthma, allergic rhinitis, autoimmune disorders such as psoriasis and rheumatoid arthritis, as well as childhood epilepsy and migraines. While some of these associations have been substantiated by meta-analyses, conventional observational studies are often challenged by selection bias, reverse causation, and residual confounding.^[13–15]^

Depression is associated with increased morbidity and mortality both in the general population and among patients with CAD.^[16]^ A significant relationship between depression and cardiac morbidity has long been recognized. The identification of depression as a negative prognostic marker in CAD during the 1990s led to hopes that treating depression could improve cardiovascular outcomes. However, these hopes have not been fully realized.^[17]^ A Cochrane review of randomized controlled trials revealed that while psychological and pharmacological interventions had a small but clinically meaningful effect on depression outcomes in CAD patients, they did not significantly reduce mortality rates or cardiac events. In some instances, treating depression even resulted in negative physical consequences.^[18–20]^ MD is one of the most prevalent mental health conditions, with a lifetime prevalence of approximately 17%.^[3,21]^ MD and cardiovascular disease are closely linked, with MD patients facing a roughly 75% increased risk of developing cardiovascular disease.^[22]^ Moreover, 30% to 74% of individuals who experience cardiac events will also meet the criteria for MD. Understanding the intricate relationship between these 2 conditions is critical for reducing premature mortality, as comorbid MD is associated with a 5-fold increase in the risk of cardiac death within 6 months following a myocardial infarction.^[23]^ In addition to the well-documented but poorly understood relationship between MD and cardiac health, emerging evidence indicates that chronic loneliness – independent of MD – also significantly elevates the risk of cardiovascular disease and early mortality.^[24]^ Analysis of nearly 500,000 participants in the UK Biobank revealed that loneliness increased the risk of cardiovascular disease by 50% and cardiovascular mortality by 30%. These findings, among others, have spurred public health initiatives to examine the physical and psychological impact of loneliness. Chronic loneliness, characterized by a perceived discrepancy between desired and actual social connectedness, affects up to 22% of adults.^[25–28]^

Mendelian randomization (MR) has emerged as a powerful tool for evaluating causal relationships between exposures and outcomes.^[29]^ By using genetic variants associated with the exposure as instrumental variables (IVs), MR helps to mitigate biases arising from confounding factors and reverse causality.^[30]^ Leveraging the extensive data available from Genome-Wide Association Studies (GWAS), our study applied a two-sample MR approach to investigate the causal relationships between MD, ADHD, and CAD.^[31]^ By analyzing large-scale GWAS data, we aimed to determine whether a genetic predisposition to MD and ADHD causally influences the risk of developing CAD. The findings of this study may offer valuable insights into the complex interplay between mental health and cardiovascular disease, potentially informing future prevention and intervention strategies.^[32,33]^

## 2. Materials and methods

### 2.1. Designing MR

**Figure 1.**
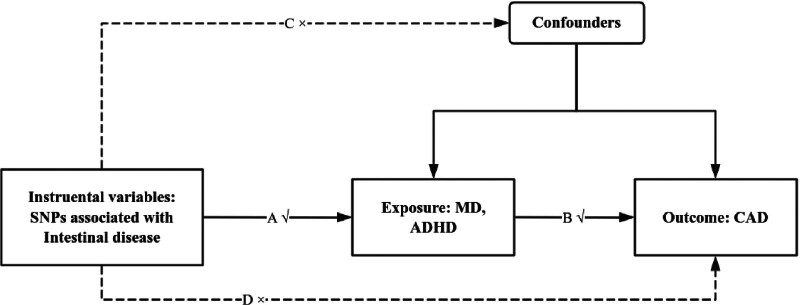
The process of MR-analysis. In this figure, the SNPs strongly associated with MD were regarded as IVs. These selected SNPs can only affect the outcomes by exposure (the solid line and arrow “A” and “B” are permitted), and all the IVs should not affect outcomes directly or through confounding factors (the dashed line and arrows from “C” and “D” are not allowed). IVs = instrumental variables, MD = major depression, MR = Mendelian randomization, SNP = single-nucleotide polymorphism.

Bidirectional MR analyses were conducted to investigate the causal effects of MD and ADHD on CAD (Fig. 1). Single-nucleotide polymorphisms (SNPs) were employed as instrumental variables (IVs) representing both the exposures (MD and ADHD) and the outcome (CAD). The heterogeneity of the IVs was assessed using Cochran *Q* statistic and the corresponding *P*-value test. Furthermore, we utilized the MR-Egger and MR-PRESSO methods to evaluate the potential for pleiotropy and to identify outliers. Sensitivity analyses were performed using the leave-one-out approach, and the results were visualized through funnel plots. It is important to note that all data and materials used in this MR study are publicly available.^[32]^

### 2.2. Criteria for selecting IVs

Instrumental variable selection criteria: In MR, IVs must meet 3 crucial assumptions^[33]^: Assumption 1: Correlation hypothesis: a robust correlation with the exposure; Assumption 2: Exclusivity hypothesis: no association with the outcome; Assumption 3: Independence hypothesis: independence from confounding factors.^[34]^ Initially, SNPs with *P* = 5 × 10^−6^, *r*^2^ = 0.001, and kb = 10000 were meticulously screened. Subsequently, the *F*-statistic for each SNP was calculated, and SNPs with *F* < 10 were excluded to mitigate the potential bias arising from weak IVs.

### 2.3. Availability of data

MD (ebi-a-GCST005903) and ADHD (ieu-a-1183) were selected as the exposure factors, with sample sizes of 217,584 and 55,374, respectively. Following the screening criteria, a total of 11 SNPs (MD) and 60 SNPs (ADHD) were identified. The outcome factor, CAD (ebi-a-GCST005195), had a sample size of (n = 547,261), 10 SNPs were selected based on the screening criteria (Table 1).

**Table 1:** Detailed information for the GWAS data.

Exposures	GWAS ID	Data source	Sample size (case/control)	PMID	Year	Population
MD	ebi-a-GCST005903	Howard DM et al	217,584 (8276/209,308)	29662059	2018	European
ADHD	ieu-a-1183	Demontis et al	55,374 (20,183/35,191)	-	2017	
Outcomes	GWAS ID	Data source	Sample size (case/control)	PMID	Year	Population
Coronary artery disease	ebi-a-GCST005195	van der Harst P et al	547,261 (122,733/424,528)	29212778	2017	European

ADHD = attention-deficit hyperactivity disorder, GWAS = genome-wide association study, MD = major depression.

### 2.4. Statistical analysis

The causal relationship between MD, ADHD, and CAD was assessed using inverse variance weighting (IVW) alongside complementary methods, including MR-Egger, weighted median, simple model, and weighted model analyses.^[35]^ The IVW estimation applied the Wald ratio method without incorporating the intercept term, using the reciprocal of the variance as the weight for linear regression fitting. Given that the selected instrumental variable SNPs satisfied the core assumptions of MR analysis, the results are considered standardized, accurate, and robust.^[35]^

To broaden the scope of genetic instruments, we adjusted the significance threshold, allowing the inclusion of genetic variants that may not fully adhere to the 3 fundamental MR assumptions. To ensure the reliability of the estimated results, sensitivity analyses were conducted using Cochran *Q* statistic to evaluate the heterogeneity of genetic variation.^[36]^ When significant heterogeneity was detected among genetic variants, a random-effects IVW model was applied; otherwise, a fixed-effects IVW model was used.

To detect potential horizontal pleiotropy among SNPs, we employed the MR-Egger method. In this analysis, the intercept term serves as an indicator of pleiotropy across all variables. If the *P*-value of the intercept term exceeds .05, pleiotropy at the genetic level is not considered present. Additionally, a leave-one-out sensitivity analysis was performed, where each SNP was systematically removed one by one, and the IVW results were recalculated. Consistency between the leave-one-out results and the main analysis suggests that no single SNP had a disproportionate influence on the outcomes.

The results are presented as odds ratios (OR) with corresponding 95% confidence intervals (CI). All analyses used two-sided *P*-values, with statistical significance set at *P* < .05. Statistical analyses were conducted using R software, specifically the “TwoSampleMR” package.^[36,37]^

## 3. Results

### 3.1. Association between MD and CAD

**Figure 2.**
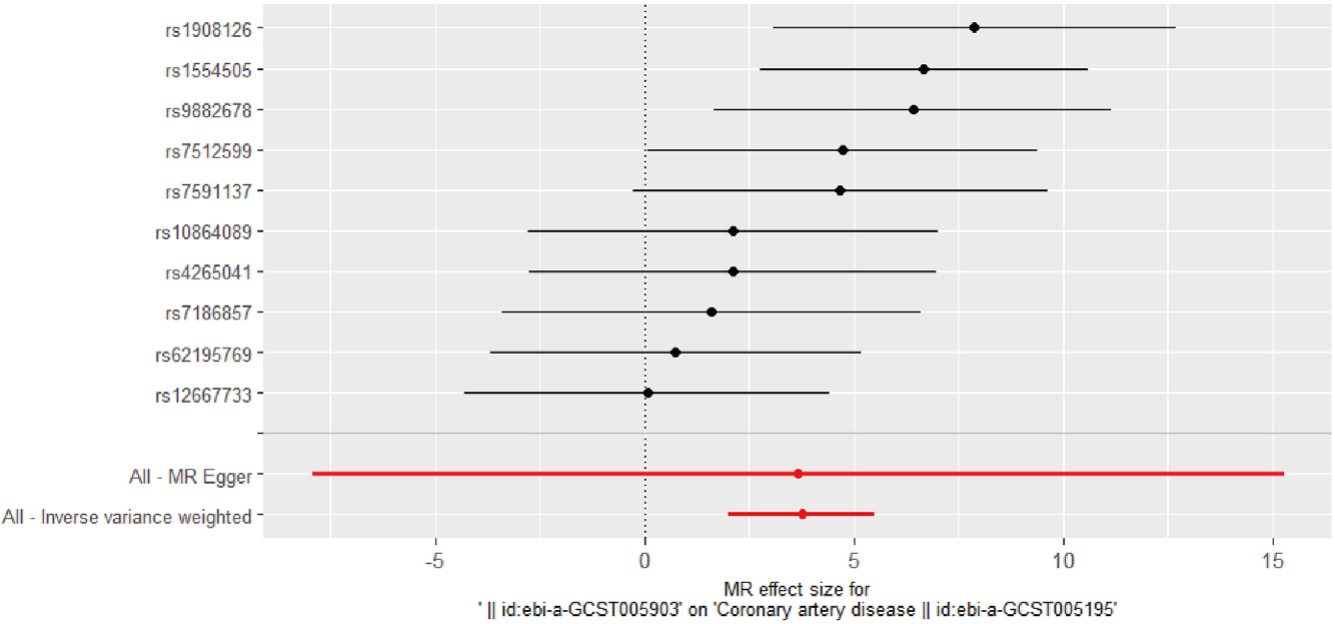
Causal relationship between major depression and CAD – forest map. CAD = coronary artery disease.

The GWAS identified 11 MD SNPs (Table S1, Supplemental Digital Content, https://links.lww.com/MD/Q344), IVW MR-analysis with a random-effects model showed a statistically significant association between MD and risk of CAD (OR: 42.66, 95% CI: 7.55–241.2; Fig. 2, Table 2). IVW regression analysis showed a significant intercept (*P* < .001, Table 3), indicating horizontal pleiotropy. Similar associations were observed with the simple mode methods (OR: 5.357, 95% CI: 0.067–427.9), with the penalized weighted median method (OR: 9.953, 95% CI: 1.011–97.99) (Table S3, Supplemental Digital Content, https://links.lww.com/MD/Q344).

**Table 2:** Two-sample Mendelian randomization for major depression disorders, attention-deficit hyperactivity disorder and coronary artery disease outcome, using inverse variant weighting.

Exposure	Disease	No. of SNPs	OR	95% CI
Major depression disorders	Coronary artery disease	11	42.66	7.55–241.2
Attention-deficit hyperactivity disorder	Coronary artery disease	60	1.055	1.006–1.106

BMI = body mass index, CI = confidence interval, OR = odds ratio, SNP = single-nucleotide polymorphism.

**Table 3:** Sensitivity analyses.

Exposures	Pleiotropy test			Heterogeneity test			
	MR-Egger			MR-Egger		IVW	
	Intercept	SE	*P*	*Q*	*Q*_pval	*Q*	*Q*_pval
Major depression disorders	0.0002	0.0203	.9898	12.6208	.1255	12.614	.1805
Attention-deficit hyperactivity disorder	0.0044	0.0079	.5837	123.306	3.252489e−09	124.140	4.172432e−09

IVW = inverse variance weighted, MR = Mendelian randomization.

### 3.2. Investigating the causal role of ADHD on CAD outcome

**Figure 3.**
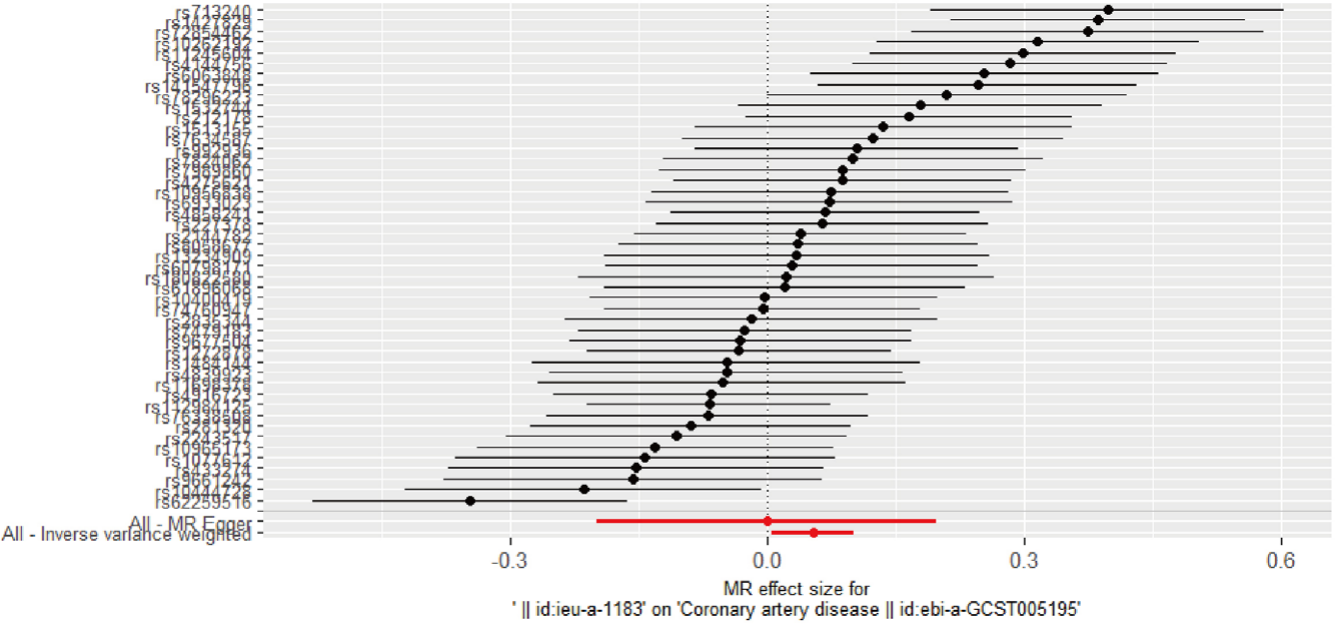
Causal relationship between ADHD and CAD – forest map. ADHD = attention-deficit hyperactivity disorder, CAD = coronary artery disease.

The MR results using the inverse variance weighting (IVW) approach are presented in Table 2. There was evidence supporting a causal effect of ADHD on CAD, with an odds ratio (OR) of 1.055 per log-odds increase in ADHD genetic liability (95% confidence interval (CI): 1.006–1.106, Fig. 3). Both the weighted median estimator and MR-Egger regression showed consistent effects in the same direction as the IVW analysis, although with wider CIs, as expected (Table S4, Supplemental Digital Content, https://links.lww.com/MD/Q344). The genome-wide association study (GWAS) identified 60 ADHD-associated SNPs (Table S2, Supplemental Digital Content, https://links.lww.com/MD/Q344).

### 3.3. Inverse variance analysis

**Figure 4.**
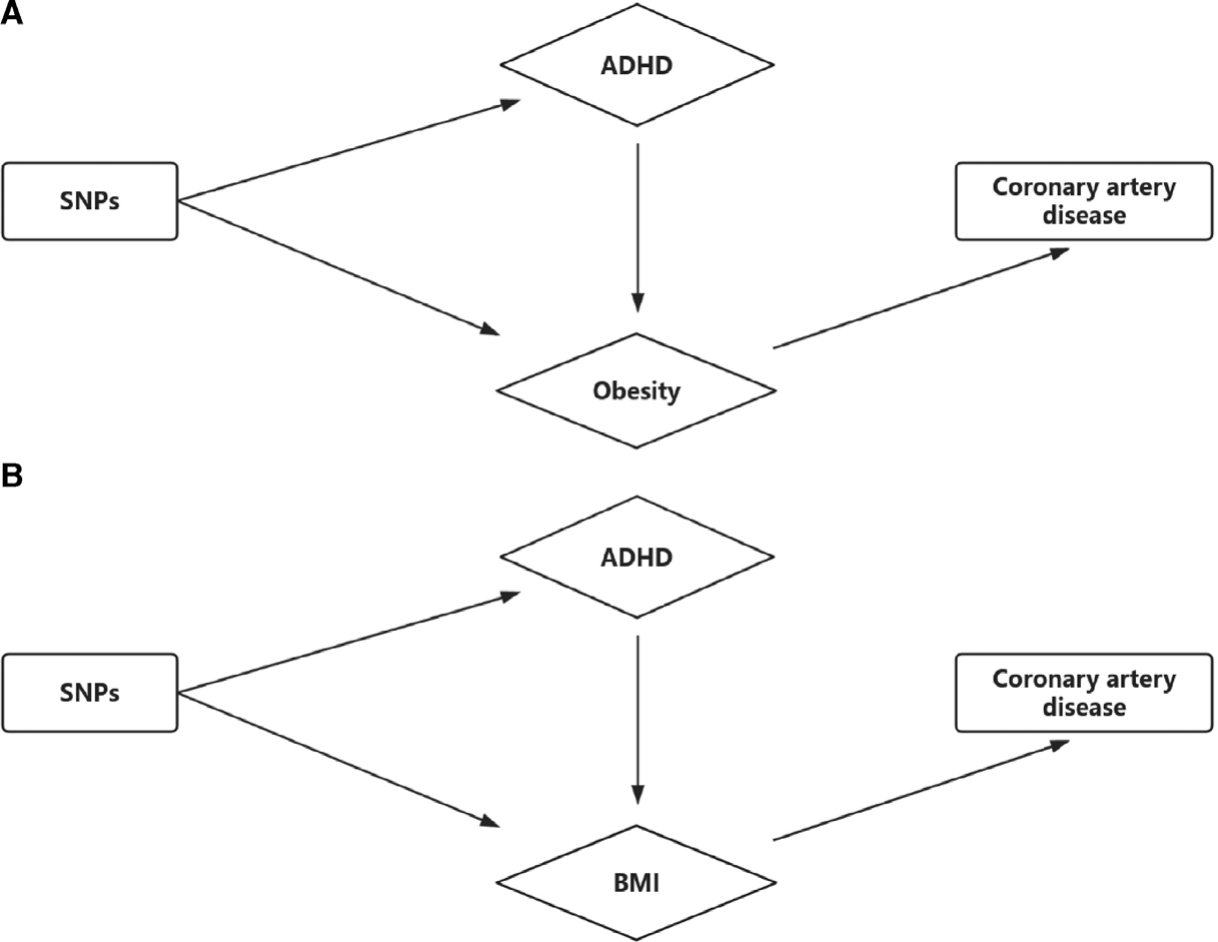
Relationships among (A) ADHD, obesity and CAD and (B) among MD, BMI according to results obtained by three-sample (univariable) MR and multivariable Mendelian randomization. (A) ADHD affects CAD through its effect on obesity, rather than through a direct effect on CAD. (B) ADHD affects CAD directly and not only through its effect on BMI. ADHD = attention-deficit hyperactivity disorder, BMI = body mass index, CAD = coronary artery disease, MD = major depression, MR = Mendelian randomization, SNP = single-nucleotide polymorphism.

We conducted bidirectional MR analyses to assess the impact of CAD and obesity on ADHD (Fig. 4). The results provided little evidence for a causal effect of CAD on ADHD, with an inverse variance weighting (IVW) estimate of 1.007 (95% CI: 0.943 to 1.076). Similarly, no evidence was found for a causal effect of obesity on ADHD, with an IVW OR of 277.69 (95% CI: 0.2506–3.08e+05). MR-Egger regression yielded directionally consistent estimates, and there was no indication of heterogeneity or horizontal pleiotropy (Table S5, Supplemental Digital Content, https://links.lww.com/MD/Q344). Additionally, there was little evidence of a causal effect of CAD on MD, with an IVW estimate of 1.002 (95% CI: 0.999–1.005). Steiger filtering did not suggest reverse causation, as all genetic instruments for ADHD explained more variance in ADHD than in CAD or obesity. Moreover, none of the 60 SNPs used as IVs for ADHD (Table S2, Supplemental Digital Content, https://links.lww.com/MD/Q344) were in linkage disequilibrium with any of the 60 SNPs associated with childhood obesity (*P* < 5 × 10^−6^), indicating no overlap between genetic instruments for ADHD and obesity.

### 3.4. Sensitivity analysis

Heterogeneity was detected in the inverse variance weighting (IVW) analysis for MD and ADHD (Table 3). However, none of the leave-one-out sensitivity analyses identified any outlying SNPs (see Figs. S1 and S2, Supplemental Digital Content, https://links.lww.com/MD/Q343). Additionally, the MR-Egger intercept did not significantly differ from zero in any of the heterogeneity tests, suggesting that the observed heterogeneity was unlikely due to bias from directional horizontal pleiotropy. Sensitivity analyses for MD and ADHD showed consistency with the results for chronic ischemic heart disease, as detailed in Table 3. In other words, all sensitivity analysis results confirmed the robustness and reliability of the MR findings.

Based on these initial findings, there was evidence of a causal effect of ADHD on obesity, CAD, and body mass index (BMI). Consequently, these 3 outcomes were subjected to further sensitivity analyses. A causal effect was detected through simulation extrapolation-adjusted MR-Egger regression for both obesity and CAD, prompting us to explore potential bidirectional effects. However, no evidence of a causal relationship was found for inflammatory bowel disease in the simulation extrapolation-adjusted MR-Egger regression, indicating pleiotropic effects, and therefore, it was not pursued in further analyses.

### 3.5. Investigating possible mediators of the association between ADHD and physical health outcomes, using MVMR

Since obesity and BMI are established risk factors for CAD^[38]^ and are strongly associated with ADHD,^[39,40]^ they may mediate the relationship between ADHD and CAD. In the multivariable Mendelian randomization (MVMR) model, when genetic variants for both ADHD and obesity were included, the direct causal effect of ADHD on CAD was attenuated to an OR of 1.02 (95% CI: 1.001–1.071), compared to the univariable MR results. Meanwhile, the effect of obesity on CAD remained stable (OR = 20.5, 95% CI: 1.91–2.2e+04) (Table S6, Supplemental Digital Content, https://links.lww.com/MD/Q344). This suggests that ADHD may contribute to CAD primarily through its influence on childhood obesity rather than directly. Therefore, there is evidence supporting the mediating role of obesity in the ADHD-CAD relationship, as illustrated in Figure 4A. The adjusted *F*-statistic was >10 in the MR-Egger analysis, indicating strong instrument validity (Table S6, Supplemental Digital Content, https://links.lww.com/MD/Q344).

Similarly, when genetic variants for ADHD and BMI were analyzed together in the MVMR model, the direct causal effect of ADHD on CAD was further reduced to an OR of 1.005 (95% CI: 0.998–1.020), while the effect of BMI on CAD remained stable (OR = 1.557, 95% CI: 1.459–1.661) (Table S7, Supplemental Digital Content, https://links.lww.com/MD/Q344). This suggests that ADHD’s impact on CAD may be mediated through its effect on childhood obesity rather than through a direct influence on CAD, further supporting the role of obesity as a mediator in this pathway, as depicted in Figure 4B.

## 4. Conclusion

The present MR study demonstrates the absence of a consistent association between MD, ADHD, and CAD. Utilizing a two-sample MR approach, we explored potential causal effects of ADHD on physical health outcomes by employing genetic variants as instrumental variables for ADHD. Our findings suggest a causal relationship between ADHD and CAD, as well as evidence of a bidirectional relationship between ADHD and obesity. However, limited evidence was found for the causal effects of ADHD on neurological, autoimmune, and allergic diseases, or lung cancer. Furthermore, multivariable MR (MVMR) results indicate that obesity may partially mediate the effect of ADHD on CAD. Individuals with ADHD are more likely to engage in behaviors such as smoking,^[40]^ experiencing weight gain,^[41–43]^ and adopting lifestyles characterized by low physical activity levels.^[44]^ Notably, results show that the impact of ADHD on CAD diminished when accounting for both ADHD and obesity, suggesting that childhood obesity may be a potential mediator. Additionally, no evidence was found for a causal effect of adult BMI on ADHD, implying that these effects may be more pronounced during childhood. Martins-Silva et al^[45]^ employed a two-sample MR approach to uncover a causal link between BMI and ADHD. This finding is particularly noteworthy because it challenges the conventional understanding that ADHD typically precedes childhood obesity. Instead, bidirectional analyses conducted by the researchers suggest the presence of dynastic effects. These effects indicate that parental genetic predispositions to certain physical health conditions, such as obesity, may not only influence their own health but also significantly increase the risk of ADHD in their offspring. This highlights the complex interplay between genetic factors and the development of ADHD, emphasizing the need for a more nuanced understanding of the disorder’s etiology.

We also observed a significant positive association between genetic liability to MD and CAD, with a suggestive association with heart failure. Reverse MR analysis did not provide evidence of an association between liability to CAD or heart failure and MD. Our findings suggest that MD may be causally linked to an increased risk of CAD and possibly heart failure, supporting previous observational studies.^[46–50]^ Wulsin et al revealed that symptoms of depression independently increase the risk of developing CAD more than exposure to passive smoking.^[51]^ Gan et al, through a meta-analysis, suggested that depression is independently associated with a significantly increased risk of CAD.^[52]^

Notably, diabetes and CAD are known risk factors for heart failure, with CAD accounting for over 60% of heart failure case.^[53]^ Thus, the observed association between MD and heart failure may be partially mediated through CAD. Several mechanisms could explain the causal effect of MD on CAD risk, including biological abnormalities linked to depression, such as increased release and activity of counter-regulatory hormones, altered glucose transport, and heightened immunoinflammatory activation, all of which may contribute to CAD risk.^[54]^ Additionally, lifestyle factors like smoking and alcohol consumption may mediate the relationship between depression and CAD.^[55]^ According to the results of a hospital-based observational study, there is a direct association between the smoking status and CAD among the young adults. In general, the incidence of CAD had a higher mean value among smokers and the age of patients was lower than or equal to 35 years old.^[51,56,57]^

Numerous studies have reported associations between ADHD and obesity during adolescence and adulthood.^[44,58–60]^ While motor hyperactivity is a hallmark of ADHD, it may seem counterintuitive that individuals with ADHD are at higher risk for obesity.^[61]^ However, observational studies have shown that individuals with ADHD tend to spend more time watching television,^[62]^ have lower levels of physical activity, and exhibit increased dysregulation of eating behaviors.^[63]^ Cardiometabolic conditions, including obesity, hypertension, type 2 diabetes, and myocardial infarctions, were more prevalent in women with a diagnosis of ADHD than in those without.^[64]^ Obesity acts as a mediating factor, playing a pivotal role in linking ADHD and CAD through various intricate biological mechanisms.^[51]^ Individuals with ADHD are predisposed to developing obesity due to behavioral traits such as impulsive eating, insufficient physical activity, and sleep disturbances.^[62,63]^ In turn, obesity heightens the risk of CAD via pathways involving chronic inflammation, metabolic dysregulation, and endocrine imbalances.^[64]^ Adipose tissue in obese individuals secretes substantial amounts of pro-inflammatory cytokines (e.g., TNF-α, IL-6, CRP), which trigger systemic chronic inflammation that compromises vascular endothelial function and promotes atherosclerosis. Concurrently, obesity leads to insulin resistance – a key contributor to metabolic syndrome – manifesting as hyperglycemia, dyslipidemia, and hypertension; all significant risk factors for CAD.^[51,52]^ Additionally, obesity induces lipid metabolism disorders characterized by elevated LDL-C levels, reduced HDL-C levels, and increased triglycerides – all exacerbating the progression of coronary atherosclerosis. Dysregulated adipokine secretion is marked by elevated leptin levels coupled with decreased adiponectin levels; this further drive vascular dysfunction and inflammatory responses. In an obese state, blood exhibits a hypercoagulable tendency due to increased fibrinogen levels and enhanced platelet activity – thereby raising the risk of thrombosis.^[60–63]^ Furthermore, obesity activates both the sympathetic nervous system and the renin-angiotensin-aldosterone system, resulting in elevated blood pressure and an augmented cardiovascular burden.^[52]^ In summary, the mediating role of obesity between ADHD and CAD underscores its multifaceted pathological mechanisms. Investigating these pathways is essential for elucidating the relationship between these two conditions while also informing effective therapeutic strategies.

## 5. Limitation

There are several key limitations in this study. Firstly, the sample size may be insufficient to detect all genetic variations associated with pulmonary embolism, especially across different races and populations. Secondly, the effectiveness of MR analysis relies on the quality and quantity of the instrumental variables selected, and we cannot completely exclude the influence of pleiotropy.

The latest ADHD GWAS was the first to identify genetic variants that are significantly associated with ADHD, but these variants still only explain little variation in the ADHD phenotype.^[64]^ To increase the number of instrumental variables for sensitivity analyses, such as MR-Egger regression, we lowered the *P*-value threshold for SNP inclusion from the ADHD GWAS to *P* < 1 × 10^−6^. This approach allowed us to incorporate additional SNPs that, while not genome-wide significant, still provided valuable information for our analyses. Although statistical evidence for horizontal pleiotropy was limited, the biological functions of many ADHD-associated variants remain unclear, and pleiotropic effects cannot be fully excluded. This study employed a two-sample MR design using publicly available GWAS data, which limited our ability to confirm the independence of instrumental variables from unmeasured confounders.^[65]^ Potential bias due to population stratification, assortative mating, or selection bias in the GWAS datasets may have influenced the observed associations. Additionally, MR estimates for binary exposures and outcomes may be subject to bias and should be interpreted with caution, focusing on the direction and magnitude of associations rather than precise effect sizes.^[66]^

Lastly, the clinical application of these findings still needs validation through large-scale cohort studies and clinical trials. Therefore, further research is needed to confirm their clinical utility before applying these findings to clinical practice.

## Acknowledgments

We would also like to express our gratitude to the Postdoctoral Program at Shandong University of Traditional Chinese Medicine for the postdoctoral fellowship and the supportive academic environment.

## Author contributions

**Conceptualization:** Xiaochen Zhang, Xiaofei Zhong.

**Data curation:** Lei Zhang, Suxia Huo.

**Formal analysis:** Xiaochen Zhang.

**Investigation:** Xiaochen Zhang, Lei Zhang.

**Methodology:** Xiaochen Zhang, Lei Zhang, Yan Lu.

**Project administration:** Xiaokai Hua, Xiaofei Zhong.

**Supervision:** Xiaofei Zhong.

**Validation:** Lei Zhang.

**Visualization:** Xiaochen Zhang.

**Writing – original draft:** Xiaochen Zhang, Suxia Huo.

**Writing – review & editing:** Lei Zhang, Xiaokai Hua, Xiaofei Zhong.

## Supplementary Material




