# Supplementary material for: The relationship between major depression, attention-deficit hyperactivity disorder and coronary artery disease: A two-sample Mendelian randomization analysis
Source: Medicine (Baltimore). 2025 Oct 17;104(42):e43188. doi: 10.1097/MD.0000000000043188 (PMC12537080; doi:10.1097/MD.0000000000043188)
Supplement: Supplementary file 1 [file medi-104-e43188-s001.docx]

**Supplementary table1. MD genetic variants associated at p<1x10^-6^**

| **chr.exposure** | **SNP** | **effect_allele.exposure** | **other_allele.exposure** | **beta.exposure** | **se.exposure** | **pval.exposure** |
| --- | --- | --- | --- | --- | --- | --- |
| 1 | rs7512599 | A | G | 0.0032002 | 0.00063782 | 5.24E-07 |
| 1 | rs10864089 | G | A | 0.0036272 | 0.00077044 | 2.50E-06 |
| 2 | rs7591137 | A | G | 0.0029312 | 0.00061909 | 2.20E-06 |
| 2 | rs62195769 | A | C | 0.0051919 | 0.00095885 | 6.15E-08 |
| 3 | rs9882678 | T | G | 0.0037374 | 0.00076738 | 1.11E-06 |
| 4 | rs115559387 | A | G | 0.011341 | 0.0023968 | 2.23E-06 |
| 4 | rs1908126 | A | G | 0.0032063 | 0.00065823 | 1.11E-06 |
| 6 | rs4265041 | A | G | 0.0032677 | 0.00068106 | 1.60E-06 |
| 7 | rs12667733 | G | A | -0.0031676 | 0.00058415 | 5.88E-08 |
| 7 | rs1554505 | A | G | 0.0040426 | 0.00067981 | 2.74E-09 |
| 16 | rs7186857 | A | C | -0.0028149 | 0.00059818 | 2.53E-06 |

**Supplementary table2. Two-sample MR for MD and CAD outcomes using 5 methods**

| **method** | **nsnp** | **b** | **se** | **pval** | **lo_ci** | **up_ci** | **or** | **or_lci95** | **or_uci95** |
| --- | --- | --- | --- | --- | --- | --- | --- | --- | --- |
| MR Egger | 10 | 3.676525 | 5.9203135 | 5.52E-01 | -7.92728993 | 15.280339 | 39.508845 | 0.000360763 | 4.33E+06 |
| Weighted median | 10 | 2.298107 | 1.1667066 | 4.89E-02 | 0.01136159 | 4.584852 | 9.955315 | 1.011426374 | 9.80E+01 |
| Inverse variance weighted | 10 | 3.753465 | 0.8835612 | 2.16E-05 | 2.0216846 | 5.485244 | 42.668654 | 7.551034733 | 2.41E+02 |
| Simple mode | 10 | 1.678574 | 2.2349445 | 4.72E-01 | -2.70191766 | 6.059065 | 5.357908 | 0.067076759 | 4.28E+02 |
| Weighted mode | 10 | 1.477304 | 2.0260192 | 4.84E-01 | -2.49369397 | 5.448301 | 4.381117 | 0.082604265 | 2.32E+02 |

**Supplementary table3. ADHD genetic variants associated at p<1x10^-6^**

| **chr.exposure** | **SNP** | **effect_allele.exposure** | **other_allele.exposure** | **se.exposure** | **beta.exposure** | **pval.exposure** |
| --- | --- | --- | --- | --- | --- | --- |
| 1 | rs112984125 | A | G | 0.0146 | -0.106005 | 3.58E-13 |
| 1 | rs9661242 | G | A | 0.0135 | 0.0625991 | 3.59E-06 |
| 1 | rs1222063 | A | G | 0.0174 | 0.0962007 | 3.07E-08 |
| 2 | rs756354 | G | C | 0.0138 | 0.0711953 | 2.46E-07 |
| 2 | rs76338508 | T | C | 0.0471 | -0.225095 | 1.78E-06 |
| 2 | rs72854462 | G | A | 0.0153 | -0.0778033 | 3.72E-07 |
| 2 | rs9677504 | A | G | 0.0206 | 0.116903 | 1.39E-08 |
| 3 | rs62259516 | T | C | 0.0286 | 0.143104 | 5.82E-07 |
| 3 | rs1513155 | A | G | 0.0182 | -0.0849045 | 2.92E-06 |
| 3 | rs4858241 | G | T | 0.014 | -0.0789036 | 1.74E-08 |
| 3 | rs4894783 | A | T | 0.0138 | -0.065798 | 1.74E-06 |
| 3 | rs7634587 | G | A | 0.0137 | -0.0635005 | 3.48E-06 |
| 3 | rs62260755 | G | C | 0.016 | 0.079996 | 5.72E-07 |
| 4 | rs227378 | A | C | 0.0143 | 0.0740958 | 2.20E-07 |
| 4 | rs1272878 | C | T | 0.0177 | -0.0910009 | 2.83E-07 |
| 4 | rs77216804 | T | A | 0.0216 | -0.103396 | 1.77E-06 |
| 4 | rs1484144 | C | T | 0.0133 | -0.0608975 | 4.27E-06 |
| 4 | rs28411770 | C | T | 0.0151 | -0.0861043 | 1.15E-08 |
| 5 | rs1077612 | T | C | 0.0165 | -0.0769026 | 3.15E-06 |
| 5 | rs433274 | C | T | 0.0199 | 0.0934978 | 2.72E-06 |
| 5 | rs1592757 | C | G | 0.0138 | 0.0729995 | 1.27E-07 |
| 5 | rs4916723 | C | A | 0.0135 | 0.0766003 | 1.58E-08 |
| 5 | rs6451675 | G | C | 0.0143 | 0.0691036 | 1.40E-06 |
| 6 | rs141547796 | A | G | 0.0256 | -0.136198 | 1.03E-07 |
| 6 | rs4839923 | A | G | 0.0133 | 0.0666956 | 5.38E-07 |
| 6 | rs6933023 | T | C | 0.0133 | 0.066602 | 5.46E-07 |
| 7 | rs180822580 | A | G | 0.0324 | -0.155298 | 1.66E-06 |
| 7 | rs10262192 | A | G | 0.0132 | 0.073204 | 2.89E-08 |
| 7 | rs28452470 | A | T | 0.0138 | 0.074003 | 8.48E-08 |
| 7 | rs13234909 | A | G | 0.0134 | 0.0627025 | 2.81E-06 |
| 8 | rs1532744 | G | A | 0.0136 | 0.0649973 | 1.64E-06 |
| 8 | rs10956838 | C | A | 0.0148 | -0.073204 | 7.60E-07 |
| 8 | rs7824062 | A | G | 0.0134 | 0.0619978 | 4.03E-06 |
| 8 | rs74760947 | G | A | 0.0317 | 0.179797 | 1.35E-08 |
| 9 | rs78296223 | T | C | 0.0289 | -0.138297 | 1.64E-06 |
| 9 | rs10965173 | G | A | 0.0167 | -0.0783027 | 2.81E-06 |
| 10 | rs713240 | T | C | 0.0133 | 0.0672006 | 4.56E-07 |
| 10 | rs9665567 | T | A | 0.0136 | -0.0696007 | 3.41E-07 |
| 10 | rs11591402 | A | T | 0.0164 | -0.0929051 | 1.34E-08 |
| 11 | rs11245604 | A | G | 0.0253 | 0.129299 | 3.30E-07 |
| 11 | rs7479183 | G | T | 0.014 | 0.0701972 | 4.98E-07 |
| 11 | rs61896068 | A | G | 0.0161 | 0.0744022 | 3.74E-06 |
| 11 | rs4275621 | G | A | 0.0137 | -0.0716972 | 1.75E-07 |
| 12 | rs1427829 | G | A | 0.0133 | -0.0799012 | 1.82E-09 |
| 12 | rs2106696 | A | T | 0.0234 | 0.107796 | 4.09E-06 |
| 12 | rs10400419 | C | T | 0.0143 | 0.0695966 | 1.15E-06 |
| 13 | rs7989860 | A | G | 0.0132 | 0.0639978 | 1.26E-06 |
| 13 | rs2243517 | C | T | 0.0136 | 0.072001 | 1.26E-07 |
| 14 | rs10444728 | A | G | 0.0139 | -0.0679042 | 9.96E-07 |
| 15 | rs60798171 | G | T | 0.0155 | 0.0733021 | 2.31E-06 |
| 15 | rs281320 | G | T | 0.0133 | 0.0737972 | 3.14E-08 |
| 16 | rs1859057 | G | C | 0.0135 | -0.0639039 | 2.10E-06 |
| 16 | rs212178 | A | G | 0.02 | -0.1154 | 7.68E-09 |
| 16 | rs8058677 | T | C | 0.0135 | 0.0680978 | 4.54E-07 |
| 18 | rs4144756 | A | G | 0.0145 | 0.0763961 | 1.48E-07 |
| 20 | rs6063848 | T | G | 0.0148 | 0.0705983 | 1.99E-06 |
| 20 | rs11698378 | C | T | 0.0175 | 0.0827948 | 2.37E-06 |
| 20 | rs2144782 | T | C | 0.0143 | 0.074003 | 2.49E-07 |
| 21 | rs992936 | C | T | 0.0135 | -0.0731018 | 5.99E-08 |
| 21 | rs2835344 | T | C | 0.0158 | -0.0754997 | 1.81E-06 |

**Supplementary table4. Two-sample MR for MD and CAD outcomes using 5 methods**

| **method** | **nsnp** | **b** | **se** | **pval** | **lo_ci** | **up_ci** | **or** | **or_lci95** | **or_uci95** |
| --- | --- | --- | --- | --- | --- | --- | --- | --- | --- |
| MR Egger | 47 | -0.000332694 | 0.10079292 | 0.997381 | -0.19788681 | 0.19722142 | 0.9996674 | 0.8204627 | 1.218014 |
| Weighted median | 47 | 0.027042647 | 0.02302532 | 0.2402051 | -0.01808698 | 0.07217228 | 1.0274116 | 0.9820756 | 1.07484 |
| Inverse variance weighted | 47 | 0.053632054 | 0.02431388 | 0.0273966 | 0.00597684 | 0.10128727 | 1.0550963 | 1.0059947 | 1.106594 |
| Simple mode | 47 | 0.009229285 | 0.04895634 | 0.8512977 | -0.08672513 | 0.1051837 | 1.009272 | 0.9169291 | 1.110915 |
| Weighted mode | 47 | -0.011797861 | 0.04789038 | 0.8065063 | -0.105663 | 0.08206728 | 0.9882715 | 0.8997278 | 1.085529 |

**Supplementary table5. Two-sample MR for obesity and ADHD outcomes using 5 methods**

| **method** | **nsnp** | **b** | **se** | **pval** | **lo_ci** | **up_ci** | **or** | **or_lci95** | **or_uci95** |
| --- | --- | --- | --- | --- | --- | --- | --- | --- | --- |
| MR Egger | 11 | -0.8147694 | 14.686675 | 0.9569707 | -29.600651 | 27.97111 | 0.4427414 | 1.40E-13 | 1.41E+12 |
| Weighted median | 11 | 4.7597097 | 4.735995 | 0.3148933 | -4.522841 | 14.04226 | 116.7120408 | 1.09E-02 | 1.25E+06 |
| Inverse variance weighted | 11 | 5.6265345 | 3.577961 | 0.1158223 | -1.386269 | 12.63934 | 277.6980746 | 2.50E-01 | 3.08E+05 |
| Simple mode | 11 | 3.7911453 | 7.247781 | 0.6123136 | -10.414506 | 17.9968 | 44.3071151 | 3.00E-05 | 6.54E+07 |
| Weighted mode | 11 | 4.1310164 | 6.055535 | 0.5106115 | -7.737832 | 15.99986 | 62.2411528 | 4.36E-04 | 8.88E+06 |

**Supplementary table6. MR for obesity and CAD outcomes using 5 methods**

| **method** | **nsnp** | **b** | **se** | **pval** | **lo_ci** | **up_ci** | **or** | **or_lci95** | **or_uci95** |
| --- | --- | --- | --- | --- | --- | --- | --- | --- | --- |
| MR Egger | 12 | 20.100018 | 9.382063 | 0.05780181 | 1.7111737 | 38.48886 | 5.36E+08 | 5.53545469 | 5.19E+16 |
| Weighted median | 12 | 6.175435 | 2.628473 | 0.01880177 | 1.0236277 | 11.32724 | 4.81E+02 | 2.78327338 | 8.31E+04 |
| Inverse variance weighted | 12 | 5.326579 | 2.387717 | 0.0256927 | 0.6466543 | 10.0065 | 2.06E+02 | 1.90914266 | 2.22E+04 |
| Simple mode | 12 | 5.952482 | 4.708781 | 0.23231575 | -3.276728 | 15.18169 | 3.85E+02 | 0.03775158 | 3.92E+06 |
| Weighted mode | 12 | 6.365185 | 3.454599 | 0.092492 | -0.4058295 | 13.1362 | 5.81E+02 | 0.6664238 | 5.07E+05 |

**Supplementary table7. MR for BMI and CAD outcomes using 5 methods**

| **method** | **nsnp** | **b** | **se** | **pval** | **lo_ci** | **up_ci** | **or** | **or_lci95** | **or_uci95** |
| --- | --- | --- | --- | --- | --- | --- | --- | --- | --- |
| MR Egger | 371 | 0.2450413 | 0.08716554 | 5.20E-03 | 0.07419683 | 0.4158857 | 1.277674 | 1.077019 | 1.515713 |
| Weighted median | 371 | 0.4323493 | 0.03826666 | 1.34E-29 | 0.35734668 | 0.507352 | 1.540873 | 1.429531 | 1.660887 |
| Inverse variance weighted | 371 | 0.4427087 | 0.03301741 | 5.40E-41 | 0.3779946 | 0.5074229 | 1.556919 | 1.459355 | 1.661005 |
| Simple mode | 371 | 0.4703159 | 0.11876855 | 8.99E-05 | 0.23752956 | 0.7031023 | 1.6005 | 1.268112 | 2.02001 |
| Weighted mode | 371 | 0.4150538 | 0.07635921 | 9.93E-08 | 0.26538979 | 0.5647179 | 1.514452 | 1.303939 | 1.758951 |
