# Supplementary material for: The relationship between major depression, attention-deficit hyperactivity disorder and coronary artery disease: A two-sample Mendelian randomization analysis
Source: Medicine (Baltimore). 2025 Oct 17;104(42):e43188. doi: 10.1097/MD.0000000000043188 (PMC12537080; doi:10.1097/MD.0000000000043188)
Supplement: Supplementary file 2 [file medi-104-e43188-s002.docx]

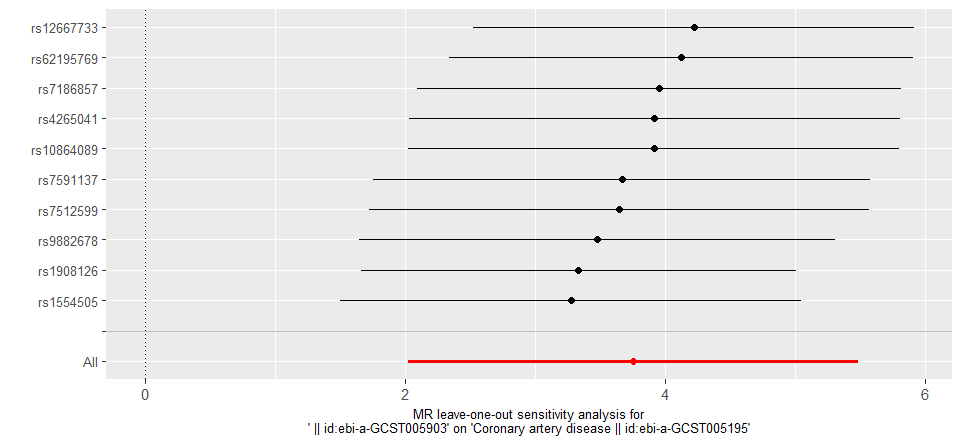


**Supplementary figure1. Causal relationship between major depression and CAD: leave-one-out map**


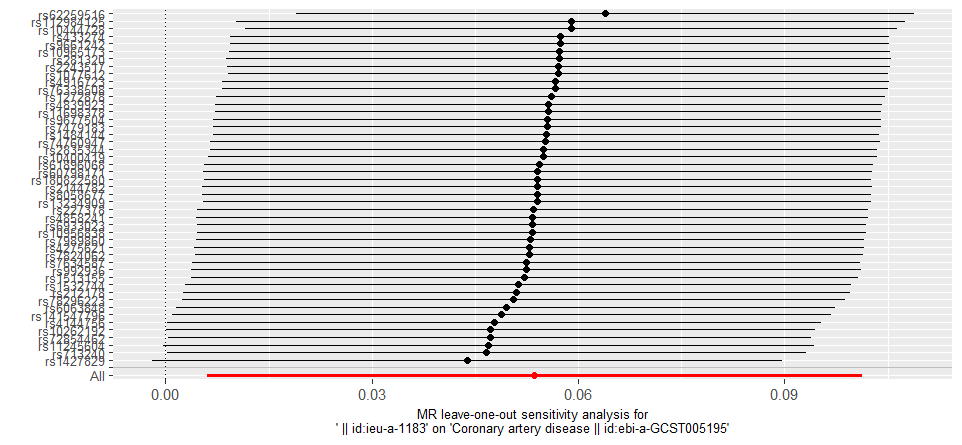


**Supplementary figure2. Causal relationship between ADHA and CAD:leave-one-out map**
